# Differences between naive and memory T cell phenotype in Malawian and UK adolescents: a role for Cytomegalovirus?

**DOI:** 10.1186/1471-2334-8-139

**Published:** 2008-10-15

**Authors:** Anne Ben-Smith, Patricia Gorak-Stolinska, Sian Floyd, Rosemary E Weir, Maeve K Lalor, Hazzie Mvula, Amelia C Crampin, Diana Wallace, Peter CL Beverley, Paul EM Fine, Hazel M Dockrell

**Affiliations:** 1 Department of Infectious and Tropical Diseases, Keppel Street, London, WC1E 7HT, UK; 2 Karonga Prevention Study, Chilumba, Malawi; 3 Department of Immunology and Molecular Pathology, UCL, London, WIT 4JF, UK; 4 The Jenner Institute, Compton, Newbury, Berkshire, RG20 7NN, UK

## Abstract

**Background:**

Differences in degree of environmental exposure to antigens in early life have been hypothesized to lead to differences in immune status in individuals from different populations, which may have implications for immune responses in later years.

**Methods:**

Venous blood from HIV-negative adolescents and blood from the umbilical cords of babies, born to HIV-negative women, post-delivery was collected and analysed using flow cytometry. T cell phenotype was determined from peripheral blood lymphocytes and cytomegalovirus (CMV) seropositivity was assessed by ELISA in adolescents.

**Results:**

HIV-negative Malawian adolescents were shown to have a lower percentage of naïve T cells (CD45RO^-^CD62L^hi ^CD11a^lo^), a higher proportion of memory T cells and a higher percentage of CD28^- ^memory (CD28^-^CD45RO^+^) T cells compared to age-matched UK adolescents. Malawian adolescents also had a lower percentage of central memory (CD45RA^-^CCR7^+^) T cells and a higher percentage of stable memory (CD45RA^+^CCR7^-^) T cells than UK adolescents. All of the adolescents tested in Malawi were seropositive for CMV (59/59), compared to 21/58 (36%) of UK adolescents. CMV seropositivity in the UK was associated with a reduced percentage of naïve T cells and an increased percentage of CD28^- ^memory T cells in the periphery. No differences in the proportions of naïve and memory T cell populations were observed in cord blood samples from the two sites.

**Conclusion:**

It is likely that these differences between Malawian and UK adolescents reflect a greater natural exposure to various infections, including CMV, in the African environment and may imply differences in the ability of these populations to induce and maintain immunological memory to vaccines and natural infections.

## Background

The immune system maintains both naïve and memory T cells, so that individuals can mount an immune response to a variety of new antigens while keeping appropriate levels of memory T cells that recognise previously encountered pathogens. Naïve and memory T cells can most simply be characterised by the reciprocal expression of the CD45RA or CD45RO isoforms [1,2]. In general, naïve (CD45RA^+^/CD45RO^-^) T cells represent the most homogeneous pool of T cells as they lack most effector functions. These cells migrate through secondary lymphoid organs seeking antigens presented by dendritic cells [3,4]. Once they encounter antigen and become activated through the T cell receptor, they proliferate and generate effector T cells that are CD45RO^+ ^with a variety of functions and that can migrate into tissues [5]. A small proportion of these effector cells persist as memory cells which give an accelerated response upon a future encounter with the specific antigen [6]. Such memory T cells can be subdivided further by their expression of the chemokine receptor CCR7 into central memory and effector memory T cells with distinct functions and homing capabilities [7].

As individuals age and encounter more new antigens, the proportion of naïve T cells declines and that of antigen-experienced memory cells increases. In older individuals, there is a generalised age-dependent decline in cell-mediated immune responses. This includes reduction in the numbers and proportions of naïve T cells, an accumulation and clonal expansion of memory and effector T cells and shrinkage of the T cell repertoire [8]. This is accompanied by an expansion of memory T cells that lack expression of the costimulatory molecule CD28 and a decrease in circulating CD28^+ ^cells [9]. Among several factors known to be associated with such changes is seropositivity to persistent viral infections such as cytomegalovirus (CMV) [10]. CMV infection is known to drive T cells toward oligoclonality, end-stage differentiation and replicative senescence [11,12], which, in elderly individuals, may contribute to increased susceptibility to infectious, neoplastic and degenerative diseases [13,14].

Comparative studies of lymphocyte phenotypes in healthy HIV-negative individuals from Ethiopia and The Netherlands [15,16], found that Ethiopians had fewer naïve T cells, and more effector and memory T cells than the Dutch. The authors concluded that the immune system of the Ethiopians was more activated compared to that of the Dutch, possibly reflecting the more frequent exposure to various infections in the African environment.

We, and others, have shown that *Mycobacterium bovis *bacille Calmette Guerin (BCG) vaccination, which is highly effective against pulmonary tuberculosis (TB) in adolescent recipients in the UK [17], provides little measurable additional protection in adults in Malawi [18] and in many other tropical countries [19]. We have also shown differences in T cell cytokine responses to mycobacterial antigens between Malawi and the UK, both before and after primary BCG vaccination, in young adults and adolescents [20]. Differences between these populations in the proportions of naive, memory and/or more differentiated memory T cells, perhaps attributable to the more frequent natural exposure to various infectious agents in Africa, may be a factor contributing to the difference in the ability of Malawians to respond to BCG vaccination. This could influence their capability to respond to other new antigens, either from infections or vaccines.

Here, we compare the proportions of naïve, memory and CD28^- ^memory (CD28^-^CD45RO^+^) T cells in adolescent and young adult HIV-negative Malawian and UK individuals, using a panel of antibodies and four-colour flow cytometry to determine the phenotype of T cells in peripheral blood. We also measured CMV seropositivity and assessed the association between anti-CMV IgG antibodies and T cell phenotype. The findings suggest that by adolescence, the Malawians have an immune system that has been challenged with more antigens including those from infections that may impact on the induction and maintenance of T cell memory.

## Methods

### Subjects

Subjects were recruited in rural northern Malawi and in suburban south east UK, between August 2002 and February 2003 and December 2002 and April 2003 respectively, in the context of a large comparative study of immune responses to BCG vaccination [20].

In Malawi, we identified adolescents and young adults with no history or evidence of BCG vaccination from records of the Karonga Prevention Study (KPS) which has been studying leprosy, tuberculosis and BCG vaccination in Karonga District since 1979 [18]. These individuals were recruited in their homes with informed, written consent from the individual or guardian, including permission to perform an HIV test. Evidence of a BCG scar, signs of TB, leprosy or other severe illness, generalised rashes, pregnancy or HIV positivity resulted in exclusion from the study. HIV serology was performed using both a particle agglutination test (Edgware modification of Serodia, Mast Diagnostics Ltd, Bootle, UK) and an ELISA (Vironostika HIV Uni-form plus 0, Organon Teknika Ltd, Cambridge, UK).

In the UK, we recruited adolescents through the schools' BCG programme operated by the Redbridge and Waltham Forest Primary Care Trusts in Essex, UK. Children born between 1988 and 1990 were invited by letter to participate in the study at the time of their routine BCG vaccination and were included after written consent from parent or guardian and verbal consent from the child, had been obtained. Exclusion criteria were evidence of previous BCG vaccination (BCG scar or vaccination records) or serious infections or immunomodulatory disease.

Fifty nine (36% male) healthy, HIV sero-negative adolescents and young adults, aged 12–25 years, with no scar or record of BCG vaccination were recruited in Malawi, and an equal number with no BCG scar or record of vaccination in the UK (age range 12–14, 47% male). The first 33 and 30 individuals recruited in Malawi and UK respectively were used to assess the proportion of naïve T cells and CD28 expression and the remaining 26 and 29 in Malawi and UK respectively, were used to examine CCR7 expression.

In both Malawi and UK, neonates were recruited during prospective cohort studies on responses to BCG vaccination in infants. Neonatal (cord blood) samples were taken following delivery of babies from HIV negative women from whom written consent had been obtained at Chilumba Rural Hospital, Karonga District, Malawi and at Whipps Cross University Hospital, Leytonstone, UK. Nineteen (47% male) and 13 (62% male) cord blood samples were collected in Malawi and the UK respectively.

Ethical approval for these studies was obtained from the National Health Sciences Research Committee in Malawi, the Redbridge and Waltham Forest Health Authority Local Research Ethics Committee, the Whipps Cross Hospital Research Ethics Committee and the Ethics Committee of the London School of Hygiene & Tropical Medicine in the UK.

### Cell preparation

Peripheral blood mononuclear cells (PBMC) were separated from 10 ml of heparinised venous or umbilical cord blood samples using Histopaque density gradient centrifugation (Sigma-Aldrich, Poole, UK) and LeucoSep^® ^tubes (Greiner Bio-One, Frickenhausen, Germany) according to the manufacturer's instructions. In the cord blood samples, red cells remaining after the Histopaque step were lysed by osmotic shock.

### Phenotyping of T cell subsets

Aliquots of 2 × 10^5 ^PBMC were stained with a panel of surface molecule-specific antibodies to the following T cell markers that were either directly conjugated to FITC, PE, PerCP or APC or biotinylated, with streptavidin-APC used as a secondary labelling reagent: CD3, CD4, CD8, CD11a, CD28, CD45RA, CD62L, CD45RO and CCR7. All the antibodies were obtained from BD Biosciences, UK. The stained cells were fixed using either CellFix (BD Biosciences, UK) in Malawi or 2% paraformaldehyde in the UK and stored at 4°C. After gating on the small lymphocyte population, 10,000–30,000 events per tube were acquired on a four-colour FACSCalibur flow cytometer (BD Biosciences, UK). Analysis was performed using CellQuest™ software. Reagents obtained from the same source and standardized protocols for cell staining, acquisition and analyses, as well as exchange of personnel, were used throughout to ensure comparability of results between Malawi and the UK. There was no evidence of differences in the percentages of small lymphocytes, CD4+ (p = 0.14) or CD8+ (p = 0.09) T cells in adolescents from the two countries (results not shown).

### Cytomegalovirus (CMV) antibody testing

Anti-CMV IgG serum antibody titres were determined for the 59 Malawian and 58/59 UK adolescents using an ELISA assay (ETI-CYTOK-G PLUS, DiaSorin, Italy) according to the manufacturer's instructions. A result above 0.4 IU/ml was taken to indicate a current or past CMV infection.

### Statistical analysis

Analysis was carried out using Stata™ 8.2 (Statacorp, USA). Histograms were plotted to examine data distributions. T tests were used to compare groups if the data were approximately normally distributed, the mean was within 3% (in absolute terms) of the median and the number of individuals in each group was at least 10. When this was not the case non-parametric tests (Wilcoxon Rank Sum Test) were used.

The effect of age was examined among Malawian individuals 12–25 years old and by comparing neonates to adolescents aged 12–15, separately for Malawi and the UK. As preliminary analyses found evidence of age-associated differences in the percentages of CD3^+^, CD4^+ ^and CD8^+ ^T cells among Malawian individuals, to allow valid comparisons between Malawi and the UK, we restricted our comparisons in the adolescent group to Malawians of a comparable age to the UK adolescents (12–15 years old) unless otherwise stated.

## Results

### Proportion of CD3^+ ^T cells that are "truly naïve"

Although naïve CD4 and CD8 T cells express CD45RA, long-term CD8 T memory cells can also re-express CD45RA [21]. "Truly naïve" cells have high expression of CD62L and low expression of LFA1 (assayed by staining for CD11a) in addition to CD45RA [22]. We used these markers to further define our naïve population as those CD4^+ ^or CD8^+ ^T cells that were CD45RO^-^, CD62L^hi ^and CD11a^lo^. The mean percentage of CD4^+ ^and CD8^+ ^T cells that were "truly naïve" was higher in the UK adolescents compared to the Malawians adolescents (p < 0.001 and p = 0.019 respectively; Table 1)

**Table 1:** Mean proportion of "truly naïve" T cells in Malawian and UK adolescents (aged 12–15 years).

	Malawi (n = 19)	UK (n = 26)	p (difference)
% CD4+ "truly naïve" (min-max)	50 (27–70) SD = 11.7	72 (55–87) SD = 7.6	<0.001

% CD8+ "truly naïve" (min max)	47 (18–71) SD = 12.9	56 (28–80) SD = 12.7	0.019

### Proportion of CD3^+ ^CD45RA^+ ^T cells that are CD28^+^

Loss of the costimulatory molecule CD28 expression in both CD4^+ ^and CD8^+ ^T cells is an indication of antigen experience [23,24]. Proportions of naïve T cells that are CD28^+ ^are greatest in neonates and decrease with age and antigen experience [25,26].

**Figure 1:**
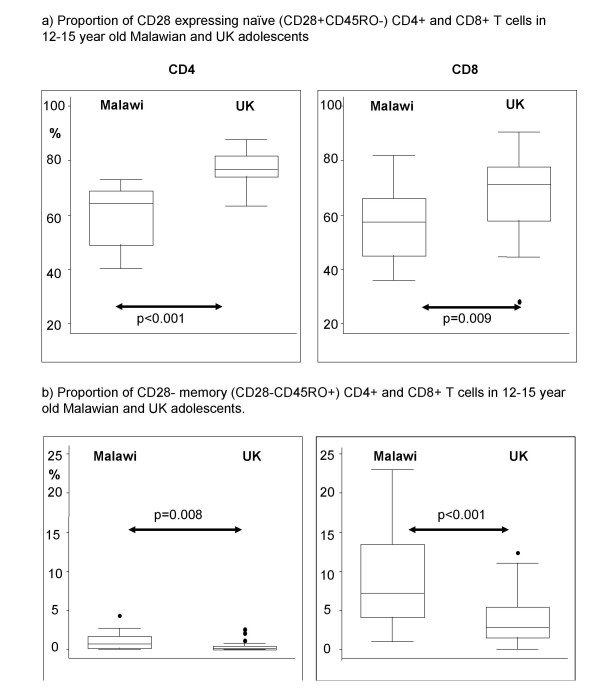
**CD28 expression in CD4^+ ^and CD8^+ ^T cells from Malawian and UK adolescents**. (a) Box and whisker plots showing proportions of CD28 expressing naive (CD28^+^CD45RO^-^) CD4^+ ^and CD8^+ ^T cells in Malawian (n = 19) and UK (n = 29) adolescents. (b) Box and whisker plots showing proportions of CD28^- ^memory (CD28^-^CD45RO^+^) CD4^+ ^and CD8^+ ^T cells in Malawian (n = 19) and UK (n = 29) adolescents. Each box shows the median and 25^th ^and 75^th ^centiles. The "whiskers" show the minimum and maximum values, other than "extreme" values that are detected as outliers and shown as circles above and below the "whiskers".

There was no evidence of a difference between the Malawian and UK neonates for the mean percentages of CD28^+ ^naïve CD4^+ ^or CD8^+ ^T cells in cord blood samples (data not shown). Adolescents in Malawi and the UK had lower mean percentages of CD4^+ ^and CD8^+ ^CD28^+ ^naive T cells compared to neonates (CD4^+ ^CD28^+ ^naïve T cells, mean of 92% in Malawian cords versus 87% in UK cords; CD8^+ ^CD28^+ ^naïve T cells, 76% compared to 79% in Malawian and UK cords, respectively). Consistent with our findings of a difference in naïve T cell proportions (as measured by CD45RA, CD11a and CD62L expression) in adolescents from the two countries, Malawian adolescents had considerably lower percentages of both CD4^+ ^and CD8^+ ^CD28^+ ^naive T cells than UK adolescents (CD4^+ ^CD28^+ ^naïve T cells, mean of 61 (range 40–73)% in Malawi versus 77 (range 63–88)% in the UK, p < 0.001; CD8^+ ^CD28^+ ^naïve T cells, mean of 56 (range 36–82)% in Malawi compared to 68 (range 28–91)% in the UK, p = 0.009)(Figure 1).

### Proportion of CD3^+ ^CD45RO^+ ^and CD45RO^- ^T cells that are CD28^-^

Consistent with the finding of fewer CD28^+ ^naïve T cells, we saw that Malawian adolescents had higher percentages of CD4^+ ^and CD8^+ ^memory T cells that lacked CD28 expression (i.e. CD28^-^CD45RO^+^) than did UK adolescents (CD4^+ ^CD28^-^CD45RO^+ ^T cells, mean of 1 (range 0–4)% in Malawi versus 0.4 (range 0–3)% in the UK, p = 0.008; CD8^+ ^CD28^-^CD45RO^+ ^T cells, mean of 9 (range 1–23)% in Malawi compared to 4 (range 0–12)% in the UK, p < 0.001). Malawian adolescents also had higher percentages of CD8^+ ^revertant memory T cells that lacked CD28 expression (i.e. CD28^-^CD45RO^-^) though the evidence for the latter difference was weak (p = 0.07, data not shown). This was apparent particularly within the CD8^+ ^T cell compartment and suggested that Malawian adolescents had been exposed to more immune stimulation than UK individuals of a similar age.

### CCR7 expression

Memory T cells can be categorised into central and effector memory T cells based on the expression of the chemokine receptor CCR7 [7]. Four T cell subsets have been described: naïve cells (CD45RA^+^CCR7^+^), central memory cells (CD45RA^-^CCR7^+^), effector memory cells (CD45RA^-^CCR7^-^) and stable or revertant memory cells (CD45RA^+^CCR7^-^). In keeping with our finding of a lower mean percentage of T cells that were naïve in Malawians, we found that, based on CCR7 expression, Malawian adolescents had lower mean percentages of naïve CD4^+ ^and CD8^+ ^T cells than UK adolescents; this was highly significant for the CD8 population, although the difference in naïve CD4^+ ^CCR7^+ ^T cells was of only borderline significance for the 12–15 year old age group (p = 0.09) becoming more significant in the older (16–25 year old) age group (p = 0.003) (see Table 2). When we examined the memory T cell phenotypes defined by CCR7 expression, it was found that, for both CD4^+ ^and CD8^+ ^T cells, Malawian adolescents had a lower percentage of central memory and a higher percentage of stable memory T cells than UK adolescents. There was no evidence of differences in the percentages of effector memory cells between the two populations.

**Table 2:** Median percentages of naïve and memory T cells based on CCR7 expression in Malawian and UK adolescents (12–15 year olds) and young adults (16–25 year olds) stratified by age.

		UK adolescents (n = 30)		Malawi adolescents whole group (n = 26)		Malawi adolescents (12-15 year olds) (n = 5)		Malawi young adults (16-25 year olds) (n = 21)	
		
		CD4^+^	CD8^+^	CD4^+^	CD8^+^	CD4^+^	CD8^+^	CD4^+^	CD8^+^
%CCR7 naïve (i.e. CD45RA^+^CCR7^+^)^a^	median	43	55	25	19	27	16	23	21
	
	range	1–79	15–87	0.4–59	1–72	24–38	9–41	0.4–59	1–72
	
	p			0.002	<0.001	0.09	0.001	0.003	0.001

%CCR7 memory that are central memory (i.e. CD45RA^-^CCR7^+^)^b^	median	47	24	14	4	9	1	14	5
	
	range	2–90	0–73	1–84	0.1–54	3–42	0.5–6	1–84	0.1–54
	
	p			0.004	0.001	0.007	0.003	0.02	0.01

%CCR7 memory that are effector memory (i.e. CD45RA^-^CCR7^-^)^c^	median	29	29	41	31	36	27	42	32
	
	range	4–50	0–56	15–76	12–56	15–41	14–34	15–76	12–56
	
	p			0.02	0.81	0.48	0.48	0.01	0.57

%CCR7 memory that are stable memory (i.e. CD45RA^+^CCR7^-^)^d^	median	18	42	35	60	52	73	27	52
	
	range	0.2–95	9–100	0.3–82	16–87	17–82	61–83	0.3–59	16–87
	
	p			0.16	0.03	0.02	0.02	0.50	0.12

P values versus UK adolescents, based on Wilcoxon rank-sum test. Ranges are minimum and maximum values.

^a ^Percentage of all CCR7 cells that are naïve.

^b, c, d ^Of the CCR7 cells that are memory, proportion that are either central memory (b), effector memory (c) or stable memory (d) (percentages in b, c, and d sum to 100).

### CMV seropositivity and T cell phenotype

As infection with CMV is associated with functional and phenotypic changes in T cells such as loss of CD28 expression and terminal differentiation, the proportion of Malawians and UK adolescents who were CMV seropositive was assessed. One hundred percent (59/59) and 36% (21/58) of Malawian and UK adolescents respectively were positive for anti-CMV IgG antibodies. The associations between CMV seropositivity and T cell phenotype, which could only be examined in the UK adolescents as all the Malawians tested were CMV seropositive are presented in Table 3. CMV seropositivity was associated with a decreased proportion of CD4^+ ^T cells and an increased proportion of CD8^+ ^T cells (data not shown); this has previously been reported in elderly seropositive individuals and is a feature of an "immune risk" phenotype associated with poor survival in old individuals [27]. CMV seropositive UK adolescents had fewer "truly naïve" CD4^+ ^and CD8^+ ^T cells compared to negative individuals; this difference reached significance in the CD8^+ ^population (p = 0.02). CMV infection was also associated with increased percentages of both CD4^+ ^and CD8^+ ^CD28^-^CD45RO^+ ^T cells. CMV seropositive individuals had a lower percentage of both CD4^+ ^and CD8^+ ^central memory (CD45RA^-^CCR7^+^) T cells compared to CMV seronegative adolescents. This could be due to a loss of central memory cells or their redistribution to lymphoid tissue but our data suggest that it is likely to be due to an accumulation of effector (CD45RA^-^CCR7^-^) and stable memory (CD45RA^+^CCR7^-^) T cells, as the proportions of both populations are raised in the CMV seropositive compared to the CMV seronegative individuals (see Table 3).

**Table 3:** Mean percentages of naïve and memory T cells in 12–15 year old CMV seronegative and seropositive UK adolescents.

		CD4+		CD8+		p value	
		
		CMV seronegative	CMV seropositive	CMV seronegative	CMV seropositive	CD4^+^	CD8^+^
		
		n = 19	n = 10	n = 19	n = 10		
%CCR7 na&#239ve (i.e. CD45RA^+^CCR7^+^)^a^	(range)	46 (2–79)	34 (1–57)	60 (43–87)	40 (15–58)	0.14	<0.001

%CCR7 memory that are central memory (i.e. CD45RA^-^CCR7^+^)^b^	(range)	54 (2–90)	41 (19–74)	32 (0–73)	14 (1–36)	0.16	0.021

%CCR7 memory that are effector memory (i.e. CD45RA^-^CCR7^-^)^c^	(range)	26 (4–45)	35 (20–50)	27 (0–48)	33 (10–56)	0.06	0.27

%CCR7 memory that are stable memory (i.e. CD45RA^+^CCR7^-^)^d^	(range)	21 (0–95)	24 (0–56)	41 (15–100)	52 (9–83)	0.72	0.21

		n = 18	n = 9	n = 15	n = 11		
		
% "truly naïve"	(range)	75 (55–87)	69 (57–77)	63 (28–80)	51 (39–66)	0.06	0.02

		n = 18	n = 11	n = 18	n = 11		
		
% CD28^- ^memory (CD28^-^CD45RO^+^) T cells	(range)	0.2 (0–0.9)	0.7 (0–2.6)	2.5 (0.4–8.1)	4.4 (0–12.4)	0.03	0.19

Ranges are minimum and maximum values.

^a ^Proportion of all CCR7 cells that are naïve. ^b, c, d ^Of the CCR7 cells that are memory, proportion that are either central memory (b), effector memory (c) or stable memory (d) (percentages in b, c and d sum to 100).

## Conclusion and discussion

In this study, we show that, at a similar age, Malawians have a lower percentage of naïve T cells and a higher percentage of CD28^- ^memory T cells than UK individuals. We also show that all our Malawian study subjects are CMV seropositive compared to only 36% of age-matched UK adolescents.

In our study, which controls for age, differences in naïve and memory T cells are shown between adolescents from Malawi and the UK and almost certainly reflect immunological experience over the lifetime of the individuals. As expected, a comparison of cord blood T cells from the two populations showed little difference in the proportion of naïve CD4^+ ^T cells. Differences in proportions of naïve and memory T cells are likely to develop early in life, as a comparative study of adults, children and neonates by Tsegaye et al [28] showed a reduction in naïve (CD45RO^-^CD27^+^) T cells before the age of 5 and suggested that this was due to antigenic challenge from birth onwards.

Malawian adolescents have proportionately fewer naïve CD4^+ ^and CD8^+ ^T cells than UK adolescents, whether naïve T cells were characterised only by expression of CD45RA (CD45RO^- ^cells) or by combinations of markers which are considered to identify "truly naïve" T cells (CD45RO^- ^with CD11a and CD62L or CD45RA with CD28 or CCR7). This is the first study to show such a difference in proportions of naïve T cells in age-matched adolescents from two countries although this has been described for elderly (>60 year olds) compared to younger individuals [24,29].

Consistent with the finding of proportionately fewer naïve T cells, Malawian adolescents have proportionately more memory (CD45RA^-^CD45RO^+^) T cells, and more differentiated memory T cells (CD28^-^CD45RO^+^) than UK adolescents. The reduction that we observe in the representation of naïve T lymphocytes with a concomitant increase in more differentiated memory T cells is similar to findings in elderly individuals [30], although it may have arisen by different mechanisms in the Malawian adolescents. The effect of a reduction in naïve T cells in the peripheral pool is the contraction of the T cell repertoire leading to poor T cell responses to new antigens (see review by Nikolich-Zugich [8]).

In a study of the effect of intestinal parasites on T cells phenotypes in Ethiopian adults, Kassu et al [31] concluded that persistent intestinal parasitic infections could result in the up regulation of T cell activation markers on CD4^+ ^and CD8^+ ^T cells, which the authors suggested would result in an imbalanced immune status which may lead to increased susceptibility to infections such as HIV. The authors showed that treatment of these various intestinal infections results in a reduction of the activated T cell phenotypes; in HIV negative individuals they showed an increase in naïve CD4^+ ^T cells, a decrease in memory/effector cells and a decrease in cytotoxic effector CD8^+ ^T cells. It may be that intestinal pathogens have far reaching effects on immune function susceptibility to disease and on responses to vaccines(as reviewed in [32]) and that their perturbation of immune responses may be reversible.

We show Malawians have proportionately fewer central memory (CD45RA^-^CCR7^+^) T cells and proportionately more CD4^+ ^and CD8^+ ^stable memory (CD45RA^+^CCR7^-^) T cells than UK subjects. This is consistent with our finding of more CD28^- ^T cells in Malawians. Gupta et al [33] showed in aged human subjects that almost all CD8^+ ^stable memory T cells and a large proportion of CD8^+ ^effector memory T cells are CD28^-^, whereas CD8^+ ^naïve (CD45RA^+^CCR7^+^) and CD8^+ ^central memory (CD45RA^-^CCR7^+^) T cells are CD28^+^. T cell recirculation and cell trafficking patterns could be affected by the presence of the large numbers of CD28^- ^T cells. Since CD28 ligation enhances the binding affinity of T cells to endothelial cells [34], the trafficking of T cells lacking CD28 between blood and tissues may be altered. Memory CD8 T cells in humans that are categorised according to the chemokine receptors (CCR7) and reacquisition of the CD45RA (naïve T cell) marker do show different homing characteristics [35].

All of the Malawian adolescents were seropositive for CMV compared to only 36% of the UK adolescents. We find that CMV seropositivity in teenagers is associated with a reduction in the percentages of naïve T cells and an increase in the proportion of CD28^- ^memory (CD28^-^CD45RO^+^) CD4^+ ^and CD8^+ ^T cells, similar to those seen in elderly CMV seropositive individuals [10] and consistent with other studies of the phenotype of CD8^+ ^T cells and CMV infection [27,36]. Furthermore, the percentages of naïve and memory T cells in CMV seropositive UK individuals are intermediate between those of CMV seronegative UK adolescents and Malawian adolescents. This is similar to differences seen between young and elderly CMV seropositive individuals, where CMV-specific CD4^+ ^T cells were found to be more differentiated in an elderly cohort compared to a young group [12]. The accumulation of effector memory (CD45RA^-^CCR7^-^) and stable memory (CD45RA^+^CCR7^-^) T cells that we see resembles the pattern which emerges when CD8^+ ^memory cells are analysed by tetramer (A2-CMV) staining [37].

The differences observed in T cell phenotypes between Malawian and UK adolescents could be due to a number of factors including; genetics, nutritional status and/or antigenic exposure due to the increased frequency of infectious diseases such as malaria, helminths and CMV. CMV seropositivity has been associated with large oligoclonal expansions of highly differentiated CD28^- ^and CD57^+ ^T cells and the presence of these differentiated T cells implies that the repertoire of both memory and naïve T cells available to respond to other antigens may be decreased [11,38]. In a study of adults (aged 19–55 years old), in seropositive individuals the percentage of human CMV specific T cells within the peripheral memory compartment was in the region of 10% for both CD4^+ ^and CD8^+ ^T cells while in seronegative individuals human CMV-specific T cells were very rare [39] suggesting human CMV is responsible for a reduction in the diversity of the T cell repertoire even in adulthood, prior to advanced age. In both young and elderly CMV seropositive donors, CMV-specific CD4^+ ^T cells were significantly more expanded and differentiated along the path to reduced replicative capacity (with loss of expression of CD27 and CD28, and shorter telomeres) than those specific for other antigens as defined by surface phenotype [12]. Moreover, in CMV-seropositive subjects, CD4^+ ^T cells of different specificities were more differentiated than the same cells in CMV-seronegative individuals, suggesting that CMV infection had a non-specific effect on the rate of differentiation of other antigen-specific CD4^+ ^T cells [12]. Taken together, our results suggest that T cells from Malawian adolescents are more highly differentiated and further down the path to reduced immune function than T cells from age-matched UK adolescents. Further studies are now needed to determine if the immune function of these more highly differentiated cells is reduced.

The reduction in naïve T cells and increase in CD28^- ^memory T cells that we observe in Malawian adolescents is also similar to changes observed and associated with the highly differentiated phenotype seen in the elderly [8]. This highly differentiated phenotype observed in the CD8^+ ^subset has been associated with CD57 expression in the CD45RA^+ ^population; some of these cells have also been shown to lack expression of CD28 [40]. Such CD28^- ^cells have been shown to have aberrant immune functions. They have a reduced ability to proliferate [25,41] and therefore cannot undergo further clonal expansions in response to antigenic stimulation. Although we did not evaluate CD57 expression in this study, if the increase in CD28^- ^T cells in Malawians was associated with increased CD57 expression, reduced functional capacity and poor proliferation in response to antigen, this might lead to ineffective control of infections.

Finally we found greater differences between Malawi and UK individuals in CD8^+ ^compared to CD4^+ ^CD28^-^CD45RO^+ ^and CCR7 memory T cells. This may be related to differences in function between CD4^+ ^and CD8^+ ^T cells, where cytotoxic (CD8^+^) T cells have more varied functions and a more diverse differentiation pattern than CD4^+ ^T cells. We are currently exploring the effector function of memory CD4^+ ^and CD8^+ ^T cell subsets in Malawi and UK individuals of a similar age using intracellular cytokine staining for IFNγ and perforin in conjunction with staining for CCR7 and activation markers. Preliminary data suggest less CD8^+ ^activation and more cell death in Malawian compared to UK T cells (P.G-S. and A.B-S. unpublished data).

Our finding of reduced naive cells and increased antigen-experienced T cells and CMV seroprevalence in young Malawians compared to age-matched UK individuals suggests that immune responses in rural African settings may be induced and maintained in a very different way than in developed countries. This may impact on both protection against infectious diseases and the ability of vaccines to induce long-lived immunity.

## Competing interests

The authors declare that they have no competing interests.

## Authors' contributions

ABS and PGS contributed equally to this work. The study was designed by HMD, PEMF, PCLB, DLW, PGS, ABS, REW and ACC. Protocols were designed by PGS, ABS, REW and ACC. Statistical analysis was done by SF. Co-ordination of the study and sample collection was carried out by PGS, ABS, REW and ACC. Immunological testing was carried out by PGS, ABS and MKL. Data interpretation and writing of the paper was done by ABS, PGS, HMD, SF, PCLB and PEMF.

## Pre-publication history

The pre-publication history for this paper can be accessed here:


